# NOD-Like Receptors in Lung Diseases

**DOI:** 10.3389/fimmu.2013.00393

**Published:** 2013-11-21

**Authors:** Catherine Chaput, Leif Erik Sander, Norbert Suttorp, Bastian Opitz

**Affiliations:** ^1^Department of Internal Medicine/Infectious Diseases and Pulmonary Medicine, Charité – Universitätsmedizin Berlin, Berlin, Germany

**Keywords:** NOD-like receptors, inflammasome, lung, pneumonia, lung injury

## Abstract

The lung is a particularly vulnerable organ at the interface of the body and the exterior environment. It is constantly exposed to microbes and particles by inhalation. The innate immune system needs to react promptly and adequately to potential dangers posed by these microbes and particles, while at the same time avoiding extensive tissue damage. Nucleotide-binding oligomerization domain-like receptors (NLRs) represent a group of key sensors for microbes and damage in the lung. As such they are important players in various infectious as well as acute and chronic sterile inflammatory diseases, such as pneumonia, chronic obstructive pulmonary disease (COPD), acute lung injury/acute respiratory distress syndrome, pneumoconiosis, and asthma. Activation of most known NLRs leads to the production and release of pro-inflammatory cytokines, and/or to the induction of cell death. We will review NLR functions in the lung during infection and sterile inflammation.

## Introduction

The respiratory tract constitutes a large surface of the body with the outside environment that is exposed to high volume airflow and large numbers of inhaled microbes and particles. The microflora of the upper respiratory tract consists of non-pathogenic bacteria but also frequently comprises potential pathogens such as *Streptococcus pneumoniae* and *Staphylococcus aureus* (1). The distal bronchi and the alveoli have long been considered sterile, however more recently microbes that are either aspirated from the upper respiratory tract (2) or constantly reside in the lower airways (3, 4) have been found by culture-independent approaches.

Inflammatory disorders of the respiratory tract involving the innate immune system include both infectious and non-infectious diseases. Lower respiratory tract infections, or pneumonia, generally develop when facultative pathogenic microbes that colonize the upper respiratory tract are aspirated or airborne pathogens are inhaled. The World Health Organization (WHO) estimates 429 million cases of acute lower respiratory tract infections in 2004, making it the third leading cause of death world-wide (5). Moreover, non-infectious and chronic lung diseases substantially contribute to morbidity. Chronic obstructive pulmonary disease (COPD) is mainly caused by tobacco smoke and can exacerbate during acute infections, ranks as the number four leading cause of death in most industrialized countries (5). Acute lung injury (ALI) and its severest form, called acute respiratory distress syndrome (ARDS), can develop after infectious as well as non-infectious insults (6). Another potentially life-threatening disorder is allergic asthma, which is characterized by airway hyperresponsiveness due to allergen-triggered airway inflammation causing chronic recurrent airflow obstruction. Long-term exposure to silica, asbestos, or coal particles can cause chronic occupational lung disease called pneumoconiosis.

The innate immune system is a key player in various infectious and non-infectious disorders of the lung (7–9). It senses infections, sterile tissue damage, and probably any disturbance of host cell and tissue integrity by so-called pattern-recognition receptors (PRRs). PRRs comprise different protein families such as the transmembrane Toll-like receptors (TLRs) and the intracellularly located nucleotide-binding oligomerization domain (NOD)-like receptors (NLRs) (10–12). PRRs recognize conserved microbial molecules, referred to as pathogen-associated molecular patterns (PAMPs) (13). However, recent evidence suggests that recognition of disturbed host cell integrity and danger signals might also play a role in the immune responses to invading pathogens (see below). In contrast to the original paradigm (13) it is now well-accepted that PRRs also sense non-microbial ligands generated during sterile tissue damage, often called damage-associated molecular patterns (DAMPs) (14, 15). Moreover, some PRRs can additionally respond to large particles and therefore appear to be key mediators in pneumoconiosis (16–18).

**Figure 1:**
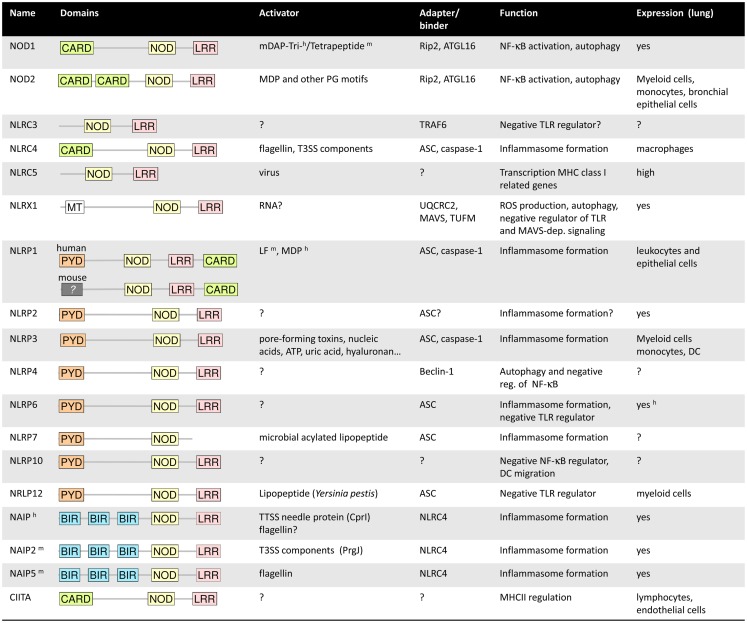
**Summary of the main characteristics of the NLRs**. ^h^ and ^m^ symbolized a characteristic specific to human or mouse. For more details, refer to the main text.

The NLR family comprises 22 members in humans and even more in mice. Most NLRs share common structural characteristics including a C-terminal leucine-rich repeat (LRR) domain, often involved in ligand recognition, a central NOD, and a variable N-terminal effector domain (10). Based on the type of effector domains that is either a caspase recruitment domain (CARD), a pyrin domain (PYD), or a baculoviral inhibitor of apoptosis protein repeat (BIR) domain, the NLR family can be further divides into five subfamilies. The NLRA subfamily consist of only one member, the transcription factor CIITA, of which at least one splice variant expresses a CARD (Figure 1). CIITA is involved in transcriptional activation of genes encoding major histocompatibility complex class II [for detailed discussion of this unique NLR protein we refer to Ref. (19)]. The NLRB group of NLRs expresses a BIR domain and consists of NAIP1–7 in mice and NAIP in humans. The NLRC subfamily includes the CARD-containing molecules NOD1, NOD2, and NLRC3–5, whereas the 14 known NLRP proteins (NLRP1–14) express a PYD. NLRX1 is the only member of the NLRX subgroup, and the only NLR protein that is localized in mitochondria (10, 20). Whereas some NLR proteins function as *bona fide* PRRs, other family members act as adaptor molecules or regulators of signal transduction.

In this review article we discuss the current knowledge about NLR expression and function in the lung in different pulmonary diseases. We have grouped the NLRs based on functional similarities and summarize major pathways and common principles of function.

## NOD1 and NOD2

NOD1 and NOD2 were the first NLR proteins to be discovered (21–25). In the lung, NOD1 is expressed in various cell types including lung epithelial cells, endothelial cells, human airway smooth muscle cells, and different types of leukocytes (26–29). NOD2 has been found in alveolar macrophages, neutrophils, and bronchial epithelial cells (30–32). NOD1 responds to bacterial cell wall peptidoglycan containing *meso*-diaminopimelic acid found predominantly in Gram-negative bacteria (33, 34). NOD2 recognizes the muramyl-dipeptide (MDP) MurNAc-l-Ala-d-isoGln, which is conserved in peptidoglycans of the majority of bacteria (35, 36). Other peptidoglycan motifs can be recognized by NOD1 and NOD2, for details refer to review (37).

Ligand recognition by both receptors leads to signal transduction through Rip2 kinase with downstream activation of MAP kinases and the transcription factor NF-κB, leading to activation of genes encoding different cytokines, chemokines (e.g., IL-8), and antimicrobial peptides. Both NOD signaling cascades are regulated by small GTPases such as Rac1, however conflicting evidence exists as to whether this regulation enhances or reduces NOD-dependent NF-κB activation (38–40). A recent study suggested that Rac1 is activated upstream of NOD1, and that NOD1 essentially senses GTPase activation rather than the peptidoglycan fragments directly (41). NOD1 and NOD2 can recruit the GTPase ATG16L1 and subsequently stimulate autophagy, a highly conserved bulk degradation system with antimicrobial activity against intracellular pathogens (42).

**Figure 2:**
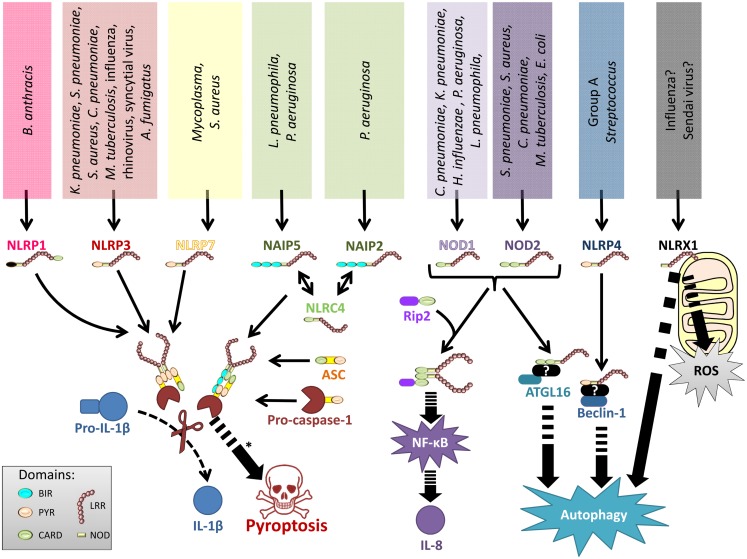
**Representation of NLRs involved in lung infections**. Various lung pathogens can be sensed by different NLR. These NLRs either form inflammasomes to regulate IL-1 family cytokines and pyroptotic cell death, stimulate production of NF-κB-dependent inflammatory mediators, regulate autophagy, or control ROS production.

Among the studied lung pathogens, NOD1 responds to *Chlamydophila pneumoniae, Legionella pneumophila, Klebsiella pneumoniae, Haemophilus influenzae*, and *Pseudomonas aeruginosa* (32, 40, 43–47), whereas NOD2 senses *S. pneumoniae, S. aureus, Escherichia coli, C. pneumoniae*, and *Mycobacterium tuberculosis* (30–32, 44, 48–50) (Figure 2). Accordingly, *Rip2^−/−^* mice – and to a lesser extend also *Nod1^−/−^* and *Nod2^−/−^* mice – display impaired chemokine production, neutrophil recruitment, and reduced antibacterial defense in response to pulmonary *C. pneumoniae* or *L. pneumophila* infection (32, 44). NOD2 is also required for efficient antibacterial innate and adaptive immunity in the chronic phase of pulmonary *M. tuberculosis* infection (51), and polymorphisms in the human *NOD2* gene have been associated with resistance or susceptibility to tuberculosis (52). Of note, mycobacteria express N-glycolylated MDP that has a stronger NOD2-activating potential compared to the MDP (53). NOD2 controls inflammatory responses to *S. aureus* pneumonia (49), and it is also required to clear pneumococcal colonization of the upper respiratory tract by CCR2-dependently recruited monocytes/macrophages. It was shown that professional phagocytes produce CCL2 after LysM-mediated bacterial digestion and subsequent NOD2-dependent detection of *S. pneumoniae*-derived peptidoglycan (48). Similarly, NOD1 controls neutrophil-dependent clearance of nasopharyngeal colonization with encapsulated *H. influenzae* in mice, whereas it is redundant for non-encapsulated strains (46). NOD1 might critically regulate microbial competition in the upper respiratory tract as *H. influenzae* derived peptidoglycan fragments activate NOD1, which instructs neutrophils to clear co-colonizing *S. pneumoniae* (54). Finally, one study implicated NOD2 in antiviral immunity to RSV and influenza virus infections (55).

Importantly, NOD proteins might also indirectly regulate immune responses in the respiratory tract. An elegant study by Weiser and colleagues showed than intestinal microbiota-derived NOD1 ligands translocate into the circulation and the bone marrow, where it enhances protective neutrophil functions in the periphery. This NOD1 induced neutrophil activation is required for efficient clearance of *S. pneumoniae* or *S. aureus* from the respiratory tract (56). NOD2 regulates the composition of the intestinal microbiota in mice (57, 58) and one might speculate about a similar function in shaping microbial communities in the upper respiratory tract.

Finally, NOD1 and NOD2 have been implicated in granulomatous and allergic lung diseases. For example, a genetic variation in *NOD1* was found to be associated with increased susceptibility to sarcoidosis in a Japanese cohort (59), and *NOD2* polymorphisms were associated with severe pulmonary sarcoidosis in Caucasian patients (60). *NOD1* as well as *NOD2* polymorphisms have been associated with increased risk of developing allergy and allergic asthma (61–64). Moreover, intranasal delivery of NOD2 ligands was shown to inhibit airway tolerance to antigens by modulating the Treg/Th2-cell balance (65). However, the function of NOD1/2 in these diseases remains ill-defined as compared to their well-established role in host defense.

## NLRP Proteins

The NLRP subgroup of NLRs comprises 14 proteins of which NLRP1, NLRP3, NLRP6, NLRP7, and NLRP12 form multiprotein complexes termed inflammasomes, consisting of one or two NLR proteins, the adapter molecule ASC and pro-caspase-1 (20). Inflammasomes serve as platforms for autocatalytic caspase-1 activation, which in turn critically regulates IL-1β and IL-18 production by processing their zymogens proIL-1β and proIL-18, and induce an inflammatory form of cell death called pyroptosis. Inflammasome activation has also been implicated in the production of eicosanoids (66). A number of NLRPs, such as NLRP6 and NLRP12 exert inflammasome-independent functions, like negative regulation of innate immune signaling pathways (as discussed below).

### NLRP1

NLRP1 was the first NLR protein to be described as forming an inflammasome (67). In humans, NLRP1 is abundantly expressed in myeloid cells, lymphocytes, and respiratory epithelial cells (68). A biochemical study showed that purified human NLRP1 can form an active inflammasome with ASC and caspase-1 in presence of MDP and ribonucleoside triphosphates (69). Nevertheless, it has so far not been clearly confirmed that MDP can trigger NLRP1 inflammasome formation in human cells.

Mice possess three genes encoding NLRP1, which are present in tandem on chromosome 11: *NLRP1a, 1b*, and *1c* (70). Depending on the genetic background, one, two, or three of these NLRP1s can be expressed. Mouse NLRP1b senses lethal toxin (LT) of *Bacillus anthracis*, leading to inflammasome activation (70, 71). Using cells from wild-type and NLRPP1^−/−^ mice, it was shown that LT but not MDP could trigger the NLRP1 inflammasome assembly (72). LT consists of two components: protective antigen (PA) and lethal factor (LF). PA mediates cytosolic uptake of LF, which has endopeptidase activity and cleaves several MAPK kinases [reviewed in Ref. (73, 74)]. This way *B. anthracis* blocks early immune responses by abrogating TLRs and NOD2 signaling (75). The expression of NLRP1b and potentially NLRP1a in macrophages, depending on the mouse background, leads to resistance to LT (70, 72, 75, 76).

It is unclear if NLRP1 mediated LT resistance exists in human cells, however it was mentioned in a recent review that the authors had never observed LT resistance in macrophages isolated from healthy human subjects (77). Studies in rats and mice, which also present cell death induced by LT depending on NLRP1, showed that LF-mediated cleavage of the N-terminal domain of NLRP1 leads to caspase-1 activation and IL-1β release (71, 78, 79). Interestingly, a recent study indicated that a direct cleavage of murine NLRP1b is sufficient to induce inflammasome activation in the absence of LF, and proposed that NLRP1 might function as a sensor of protease activity of multiple pathogens (79).

### NLRP3

Expression of NLRP3 is strongly induced by inflammatory cytokines and TLR agonists in myeloid cells (68, 80). Moreover, low level expression has also been found in human bronchial epithelial cells (81). Similar to the other inflammasomes, the NLRP3 inflammasome mediates caspase-1-dependent processing of proIL-1β as well as proIL-18 into their mature forms and stimulates pyroptosis (20).

The NLRP3 inflammasome responds to a broad range of microbial and non-microbial agents. Among lung pathogenic microorganisms, *K. pneumoniae, S. pneumoniae, S. aureus, C. pneumoniae, M. tuberculosis, L. pneumophila*, influenza virus, human rhinovirus, RSV, and *Aspergillus fumigatus* have been shown to induce NLRP3 activation (82–99). It is generally accepted that those microbes or their molecules do not directly interact with NLRP3, but instead microbe-induced disruption of host cell physiology is sensed by NLRP3. The exact nature of the NLRP3 activating signal remains somewhat elusive although production of reactive oxygen species (ROS) (16, 100), mitochondrial dysfunction (101, 102), potassium efflux (103, 104), calcium mobilization (105), have been implicated in NLRP3 inflammasome activation during infection. Most pathogens stimulate ROS production in host phagocytes, which might be involved in NLRP3 activation (85, 93). Furthermore, *S. aureus, S. pneumoniae, M. tuberculosis*, and influenza virus disturb the cell membrane and/or the intracellular ionic concentrations by their pore-forming toxins, secretion apparatus or ion channel proteins (82, 86–88, 96, 106). Other pathogens might activate NLRP3 through an incompletely defined mechanism upstream of NLRP3 that senses microbial RNA (107), and Gram-negative bacteria stimulate a non-canonical caspase-11 inflammasome (108–111). Interestingly, NLRP3 inflammasome activation by non-pathogenic bacteria that do not actively disrupt host cell integrity is dependent on bacterial viability. Live but not dead bacteria contain significant amounts of mRNA, the recognition of which triggers NLRP3 inflammasome formation. This response requires the adaptor protein TRIF, but it remains unclear whether prokaryotic mRNA can directly activate NLRP3 or if it is the result of a proximal signaling event. Detection of bacterial mRNA is a key mechanism employed by the host immune system to sense the presence of viable and thus infectious microbes and thereby to scale the level of infectious threat (112, 113). These findings underscore the role of NLRP3 as a sensor of microbial (and non-microbial) danger signals.

Several *in vivo* infection models have highlighted the central role of NLRP3 in host defense. NLRP3 was required for efficient antimicrobial responses against *S. pneumoniae, K. pneumoniae*, and influenza A virus *in vivo* (84, 85, 87, 88). Interestingly, the known susceptibility of aged mice toward influenza infection has been attributed to a reduced expression of NLRP3, ASC, and caspase-1 (114). It remains to be studied whether a similar mechanism contributes to the elevated susceptibility of elder humans to community-acquired pneumonia (115). Importantly, NLRP3 activation may also contribute to ALI, which was observed in a mouse model of *S. aureus* pneumonia (96). The net effect of NLRP3 during pneumonia might thus dependent on the pathogen load, the virulence of the pathogen and/or the expression of inflammasome components, as well as the susceptibility of the patient to pulmonary damage.

Of note, NLRP3 (and even more pronounced the NLRP6; see below) inflammasome activation in the gut shapes the intestinal microbiota (116). The commensal microflora in turn induces expression of NLRP3, proIL-1β, and proIL-18 in the lung (117). This microflora driven host gene regulation is beneficial since antibiotic depletion of the resident microbiota resulted in markedly elevated susceptibility to influenza A virus infection in mice (117).

Importantly, the NLRP3 inflammasome responds to a vast range of sterile stimuli, particularly so-called DAMPs released by dying cells including ATP, uric acid metabolites, biglycan as well as hyaluronan (106, 118–121). Experimental studies in mice suggest activation of NLRP3 by some of those DAMPs might have important functions in the pathogenesis of ALI/ARDS, COPD/emphysema, and lung fibrosis.

Efficient pulmonary gas exchange critically depends on the integrity of the fragile lung barrier composed of the alveolar epithelium and the endothelium of the pulmonary microvasculature. ALI and ARDS can develop in the course of pneumonia, sepsis, as a result of mechanical ventilation and hyperoxia, aspiration of gastric content, or major trauma (6). ALI and ARDS are characterized by a disrupted lung barrier, resulting in interstitial and alveolar edema, impaired gas exchange, and in severe cases organ failure and death. In addition, lung fibrosis may develop as a long term consequence of ALI/ARDS. Bleomycin treatment as a mouse model of acute inflammation and fibrosis results in uric acid- and ATP-release by dying cells that stimulated NLRP3 activation and IL-1β production and IL-1R-mediated inflammation, remodeling, and fibrosis (122–124). Bleomycin induced inflammation and fibrosis can be rescued by treatment with IL-1R antagonist (Anakinra) (123), allopurinol (impairs uric acid synthesis), uricase (degrades uric acid) (122), and apyrase (degrades ATP) (124). Moreover, it has been suggested that hyperoxia leads to NLRP3 inflammasome activation, secretion of pro-inflammatory cytokines, epithelial barrier dysfunction, and cell death (125, 126). Mechanical ventilation was shown to enhance IL-18 levels in the lung and serum, and inhibition of caspase-1 or IL-18 reduced ventilation-induced lung injury (127). Human ARDS patients express increased mRNA levels of inflammasome-related genes and IL-18 protein in their peripheral blood (127).

NLRP3 inflammasomes might also contribute to pathogenesis of chronic pulmonary disorders such as COPD and emphysema. Concentrations of uric acid is increased in broncho-alveolar fluid (BALF) of smokers and individuals with COPD as compared to healthy controls (128). COPD patients also have reduced levels of IL-1R antagonist (IL1RA) compared to controls (129). The mouse model of elastase-induced emphysema depends on uric acid, NLRP3, ASC, IL-1R, and MyD88 as critical mediators of inflammation, alveolar wall destruction, and fibrosis (130). Conflicting data exist regarding the contribution of the NLRP3 pathway in tobacco smoke-induced pulmonary inflammation. Whereas the study by Doz et al. indicated that smoke-induced inflammation is mediated by TLRs, the purinergic receptor P2X7, caspase-1, and IL-1R (131, 132), Pauwels et al. reported in another study that smoke-induced pulmonary inflammation occurs independently of the NLRP3 inflammasome (133). Transgenic overexpression of mature IL-1β in the lung epithelium of mice evokes a phenotype that closely resembles COPD, including inflammation, emphysema, airway fibrosis, and mucus cell metaplasia (134). Finally, *H. influenzae* infection induces NLRP3 expression and activation in human lung tissue, which might be a mechanism of infection-triggered COPD exacerbations (135). These studies together indicate an important role of caspase-1 and IL-1β in COPD and emphysema.

Conflicting evidence exists regarding the role of NLRP3 inflammasome-dependent IL-1β production in experimental asthma. Whereas ovalbumin-induced airway inflammation requires NLRP3 and IL-1β, house dust mite allergens induce pathology in an NLRP3-independent fashion (136, 137).

Pneumoconiosis is an occupational lung disease resulting from long-term exposure to silica, asbestos, or coal particles. It is characterized by pulmonary inflammation as well as fibrosis, which may be driven by NLRP3 inflammasome activation. It has been shown that engulfment of silica or asbestos crystals by resident macrophages leads to NLRP3 inflammasome activation and IL-1β production (16–18). It was suggested that crystal-induced inflammasome formation is a consequence of phagolysosomal disruption and leakage of enzymes such as cathepsin B into the cytoplasm (18). *Nlrp3^−^/^−^* and *Asc^−^/^−^* mice are protected from silica or asbestos-induced granuloma formation and fibrosis (16, 17). In contrast, mesothelioma development, a serious long term consequence of asbestosis, appears to be independent of NLRP3 (138). A recent case-control study in a Chinese population suggested that a NLRP3 polymorphisms may confer increased risk for coal workers’ pneumoconiosis (139).

Taken together, NLRP3 is a key sensor of disturbed cell and tissue integrity during infectious and non-infectious pulmonary disorders.

### NLRP4

NLRP4 has been proposed to be involved in reproduction in mammals (140–142). Nevertheless, NLRP4 expression in humans is found in various organs including the lung (142–144). *In vitro*, this NLR has the feature of a negative regulator of inflammatory responses by lowering NF-κB activation and IFNβ production (143, 144). Another particularity of NLRP4 is that its PYD is structurally different compared to the one in other NLRs, leading to the absence of interaction of NLRP4 with ASC (145). Besides, it has been described that NLRP4 negatively controls autophagy during group A streptococcal infection by interacting with the autophagy regulator Beclin-1 (146). However, in the absence of conditional gene-targeted mice it is hard to predict a functional contribution of NLRP4 to pathologies in the lung.

### NLRP6

NLRP6 has been indicated to fulfill anti-inflammatory functions by inhibiting NF-κB signaling downstream of, e.g., TLRs in macrophages and mouse (147). Moreover, elegant studies by the Flavell’s laboratory showed that NLRP6 can form an inflammasome in intestinal epithelial cells that appears to sense components of the gut microflora and in turn regulates the composition of this flora through IL-18 (116). Related or unrelated to these mechanisms, NLRP6 has also been implicated in wound healing of the intestinal mucosa (148). NLRP6 have so far been mainly described in intestinal epithelial cells, neutrophils, and macrophages (116, 147) but our own unpublished data show expression of this protein also in activated murine alveolar epithelial cells (data not shown). The function of NLRP6 in the lung has, however, not been studied yet.

### NLRP7

The NLRP7 gene is only present in humans and it is expressed in peripheral blood mononuclear cells (PBMCs) upon LPS and IL-1β stimulation (149). Gene silencing experiments in human monocytes and macrophages recently indicated that NLRP7 responds to bacterial lipopeptides and *Mycoplasma* as well as *S. aureus* infections by forming an inflammasome (150). Its precise function during bacterial infections remains unknown, and no data is available regarding its role in pulmonary physiology.

### NLRP12

NLRP12 is expressed mainly in myeloid cells (151–153). Its expression is reduced by TLR stimulation and TNFα (151, 154) NLRP12 has been described as a negative regulator of classical and non-classical NF-κB activation downstream of TLR or cytokine receptors, by interacting with IKK and NIK (151, 155, 156). Furthermore, NLRP12 has been indicated to form an inflammasome, however this has so far only been observed upon *Yersinia pestis* infection (157). Finally, NLRP12 might play a role in adaptive immunity by controlling migration of DCs to the draining lymph nodes (153). *NLRP12^−/−^* mice did not respond differently to *M. tuberculosis* and *K. pneumoniae* lung infections and allergic airway inflammation wild-type mice in (158, 159), suggesting a functional redundancy with other NLRs, or a minor contribution of NLRP12 to inflammatory processes in the lung.

## NLRC4 and NAIP Proteins

NAIP5 and NLRC4 are expressed in the cytosol of bone-marrow and alveolar macrophages. A polymorphism in *NAIP5* (also called Bircle) has long been known to affect resistance of inbred mice toward *L. pneumophila* (160, 161). Whereas most mice strains are resistant against *L. pneumophila* infection due to a functional NAIP5, A/J mice expressing a NAIP5 that differs in 14 amino acids or *NAIP5^−/−^* mice allow *L. pneumophila* replication (162–164). This NAIP5-mediated resistance against *L. pneumophila* is dependent on detection of flagellin (162, 165), and on pyroptosis of the infected macrophage as well as effects on the trafficking of the *Legionella*-containing vacuole (166, 167). Similarly, NLRC4 is well known for mediating caspase-1-dependent responses to *L. pneumophila* and other flagellated bacteria (168–171). NLRC4, however, also respond to bacteria that express a type 3 secretion system (T3SS) including, for example, *P. aeruginosa* (172–176). Of note, the *Pseudomonas* T3SS effector protein ExoU can inhibit this inflammasome activation (176). One study suggested that NLCR4 is partially involved in the production of IL-1β and inflammasome-independent cytokines upon *K. pneumoniae* infection *in vivo* (177), even though *K. pneumoniae* does neither express flagella nor T3SS.

It is now clear that murine NLRC4 forms together with either NAIP5 (and possibly NAIP6) or NAIP2 two different inflammasomes that recognize flagellin or T3SS rod proteins, respectively (178, 179). These inflammasomes appear to regulate IL-1β/IL-18 through ASC and pyroptosis independently of ASC (175, 180, 181).

The exact function of human NAIP is currently incompletely understood. We and others suggested that hNAIP can detect and restrict flagellated *Legionella* (182–184), whereas others indicated recognition of bacterial T3SS needle proteins by hNAIP (178).

## NLRC3 and 5

NLRC3 is expressed in macrophages as well as lymphocytes and has been suggested to function as a negative regulator of the early TLR signaling (185). NLRC5 is highly expressed in mouse and human lung tissue. Its expression can be further induced by IFNγ and LPS in macrophages (186–189). NLRC5 protein has been described to inhibit IL-1β, TNFα, and IL-6 productions in macrophages upon viral infection. Its most prominent role is probably in adaptive immunity by enhancing the transcription of MHC class I related genes (186, 187, 189–197). In light of these data, NLRC5 involvement in pulmonary infection such as influenza infection will be an interesting area of study.

## NLRX1

NLRX1 is ubiquitously expressed and located at the mitochondria due to an N-terminal mitochondrial targeting sequence, although the precise location (matrix or outer membrane) is still controversial (198, 199). The C-terminal LRR domain has been shown to bind to RNA but not to DNA by (200). Silencing of NLRX1 expression or knockout at the exons 4–5 in mice leads to exacerbated immune responses *in vivo* upon TLR stimulation and influenza or Sendai virus infections (201, 202). Knockout of the first four exons or exon 3, however, had no influence on the immune response to Sendai and influenza virus infections (203, 204). Instead, NLRX1 might function as an inducer of mitochondrial ROS production (205). This is consistent with the finding of two different groups that NLRX1 interacts with a protein of the mitochondrial respiratory chain (198, 203). Finally, NLRX1 has been indicated to regulate autophagy (206). Further work is clearly needed to clarify the function and exact mode of action of this unique NLR protein in general and in the lung in particular.

## Concluding Remarks

Nucleotide-binding oligomerization domain-like receptors proteins are without doubt key players in the innate immune responses to infectious and sterile inflammatory diseases of the lung, although many functions of several NLR family members, particularly in the lung, are still unknown. Many NLRs respond in functional cooperation with other innate sensors to invading microbes, particles, and endogenous danger signals after tissue damage. In similarity to possibly most immune receptors they can exert beneficial or detrimental functions, depending on the magnitude and the context of their activation. Increasing knowledge on specific activators and inhibitors of these pathways might help to manipulate them therapeutically in the not-so-distant future.

## Conflict of Interest Statement

The authors declare that the research was conducted in the absence of any commercial or financial relationships that could be construed as a potential conflict of interest.
